# Sexually transmitted infections and pre-exposure prophylaxis: challenges and opportunities among men who have sex with men in the US

**DOI:** 10.1186/s12981-016-0089-8

**Published:** 2016-01-19

**Authors:** Hyman M. Scott, Jeffrey D. Klausner

**Affiliations:** Bridge HIV, San Francisco Department of Public Health, San Francisco, CA USA; Division of Infectious Diseases: Global Health, Department of Medicine, University of California, Los Angeles, Los Angeles, CA USA

**Keywords:** STIs, HIV, PrEP, MSM

## Abstract

Pre-Exposure Prophylaxis (PrEP) has shown high efficacy in preventing human immunodeficiency virus (HIV) infection among men who have sex with men (MSM) in several large clinical trials, and more recently in “real world” reports of clinical implementation and a PrEP demonstration project. Those studies also demonstrated high bacterial sexually transmitted infection (STI) incidence and raised the discussion of how PrEP may impact STI control efforts, especially in the setting of increasing *Neisseria gonorrhoeae* antimicrobial resistance and the increase in syphilis cases among MSM. Here, we discuss STIs as a driver of HIV transmission risk among MSM, and the potential opportunities and challenges for STI control afforded by expanded PrEP implementation among high-risk MSM.

## Background

There have been exciting recent advances in human immunodeficiency virus (HIV) prevention for men who have sex with men (MSM) in the last several years, especially favorable results of three large clinical trials of Pre-Exposure Prophylaxis (PrEP) for HIV prevention [1–3]. The implementation of PrEP, and other HIV and sexually transmitted infection (STI) prevention strategies, is essential to reduce the estimated 30,000 new HIV diagnoses that occur among MSM in the US each year—with special attention to young MSM and MSM of color, groups disproportionally impacted by HIV infection [4, 5].

New strategies are also needed to address the rise in bacterial sexually transmitted infections (*Neisseria gonorrhea, Chlamydia trachomatis,* and syphilis) as well among MSM in the US [6, 7]. Syphilis cases are of particular concern given the 47.9 % increase in diagnoses between 2010 and 2014. With that increase in cases, several jurisdictions have also noted a rise in more severe syphilis complications, including a recent cluster of 18 ocular syphilis cases in San Francisco, California and Seattle, Washington—four of which resulted in at least partial visual loss, and two of which are legally blind after 5 months of follow-up [8].

The convergence of PrEP as a new HIV prevention strategy with an ongoing increase in STIs, has sparked important discussion about STI control challenges and opportunities in the setting of PrEP implementation among MSM [9].

### STIs as a driver of HIV incidence among MSM

Bacterial STIs have received significant attention as a potential driver of HIV infection among MSM since the beginning of the HIV epidemic [10, 11]. Several cohort studies of MSM initially identified a self-reported history of gonorrhea as an independent risk factor for HIV seroconversion, and both ulcerative and non-ulcerative STIs have been associated both epidemiologically and biologically with HIV infection [12–20]. Rectal STIs in particular have been identified as increasing the risk for HIV seroconversion, likely secondary to the higher per-contact transmission risk of HIV acquisition in condomless receptive anal intercourse due to the pro-inflammatory micro-environment, recruitment in HIV target cells, and mucosal immune cell activation [21]. A retrospective cohort of MSM demonstrated that two or more prior rectal gonococcal or chlamydial infections were associated with 8 times greater risk of HIV seroconversion [14]. Furthermore, a recent modeling analyses estimated the independent contribution of anorectal gonococcal or chlamydial infection on HIV incidence at approximately 15 % [22].

However, the challenge of quantifying the independent risk of bacterial STIs on HIV seroconversion risk has been confounded by sexual risk behavior. In an interesting analysis, Kelley et al., used propensity scores to control for confounding by behavioral risk and demonstrated a nearly threefold increased risk of HIV (Hazard ratio 2.7; 95 % Confidence interval (CI): 1.2–6.4) among MSM diagnosed with rectal gonorrhea or chlamydia in their cohort [23]. Syphilis was not associated with HIV acquisition, likely due to the low number of cases. A recent report from a cohort of MSM in San Diego diagnosed with recent or acute HIV infection between 2004 and 2014, did however identify syphilis as an independent predictor of HIV acquisition [24]. Other studies have also demonstrated a strong, independent association between recent syphilis infection and HIV acquisition [25].

While the increased risk of HIV acquisition with rectal STIs and syphilis has been demonstrated in controlled studies, it has been more difficult to demonstrate in “real world” settings. First, HIV and STI testing frequency is highly variable for MSM for a variety of patient and provider factors [26, 27]. Because the majority of STIs are asymptomatic in the absence of frequent screening the majority will be undiagnosed. In addition, the development of community norms regarding non-condom sexual risk reduction strategies can potentially decrease HIV risk, and concurrently increase STI risk [28]. For example, as early as 2006 in San Francisco, there was an increase in STI cases without a concurrent increase in HIV [29]. Seroadaptive sexual behaviors (e.g., serosorting or strategic positioning) were considered as a possible non-condom-related HIV risk reduction strategy employed by MSM to explain that discrepant observation [30, 31]. While seroadaptive behaviors have been associated with slightly reduced HIV risk (likely secondary to the associated difference lower per-contact risk of specific activities), they may increase risk for other STIs.

### PrEP clinical trials among MSM show high HIV risk reduction and STI incidence

Several large clinical trials have studied the efficacy of PrEP for primary HIV prevention among MSM. The Pre-Exposure Prophylaxis initiative (iPrEx) was the first large efficacy trial to show the efficacy of co-formulated tenofovir disoproxil fumarate/emtricitabine for primary HIV prevention among MSM [1]. Overall efficacy was 44 %, but was significantly higher among those with detectable drug levels indicating higher levels of adherence. Sexually transmitted infection incidence was also high in iPrEX, and a subsequent analysis showed a trend toward decreased reduction in protection among participants diagnosed with syphilis, although this was not statistically significant [25].

Results from other recent PrEP clinical trials have also demonstrated high STI incidence among study participants. The PROUD study demonstrated 86 % reduction in HIV risk among high-risk MSM offered PrEP as part of routine care at sexual health clinics in England [2]. Originally designed as a pilot study the HIV incidence among those in the delayed arm was 8.9/100 person-years. The study was able to target the highest risk men, as evidenced by both the high HIV incidence in the delayed study arm and that approximately 50 % of the cohort was diagnosed with at least one bacterial STI in follow-up. The Ipergay study also showed high PrEP efficacy, 86 % risk reduction when used for event-level instead of daily dosing, among high-risk MSM in Canada and France [32]. One third of study participants in the Ipergay study were diagnosed with an STI in follow-up, including six cases of hepatitis C (although it’s not clear how many of those were sexually transmitted).

### STI incidence is also high among PrEP users in “real world” settings

Given the high STI incidence observed in PrEP clinical trials, there has been concern about additional risk compensation—increased sexual risk behavior—in “real world” settings which has the potential to worsen the rising bacterial STI incidence. While there were no increases in sexual risk behavior overall in a recent PrEP demonstration project and report of PrEP implementation in a large healthcare system, some PrEP users did report an increase in risk behaviors, including a decrease in consistent condom use [28, 33]. In those recent reports approximately 50 % of participants were diagnosed with an STI within 12 months of follow-up. The high STI incidence in those studies, suggest that PrEP uptake occurred among the men at highest risk for HIV acquisition, but without a comparison group it is not possible to identify the role that PrEP may have played in modifying sexual risk behavior, if any, in the high STI incidence. Those studies also highlight the efficacy of PrEP in “real world” use as there were no HIV infections among the participants in the Kaiser San Francisco cohort (n = 657), and only two HIV seroconversions among 557 participants in the PrEP Demonstration Project.

### Drug resistant gonorrhea is of public health importance

*Neisseria gonorrhoeae* antimicrobial resistance is of urgent public health importance as *N. gonorrhoeae* have developed resistance to all currently available antibiotic regimens [34]. The current Centers for Disease Control and Prevention (CDC) recommendations for gonorrhea treatment include the use of dual therapy with two antimicrobials [35]. Resistance has developed to all classes of antibiotics and there is concern about further spread of multi-drug resistant clones among MSM, a core group for drug resistant gonorrhea transmission [36]. After a brief decline in 2013, the proportion of gonococcal isolates that had an elevated ceftriaxone minimal inhibitory concentration (MIC) (>0.125 µg/ml) in the Gonococcal Isolate Surveillance Project increased in 2014 [37]. There have also been reported cases of isolates with even higher ceftriaxone MICs (>0.5 µg/ml) and high-level azithromycin resistance [38, 39].

Increased STI screening and treatment will have important implications for STI control strategies to reduce the frequency of drug resistant gonococcal strains. Oropharyngeal gonococcal infections are an important source of ongoing gonorrhea transmission and also provide a microbiological environment conducive to the development of extended-spectrum cephalosporin resistance, through gene transfer with commensal *Neisseria* species that normally reside in the throat [40]. With improved screening frequency at extragenital sites like the oropharynx or rectum using more sensitive nucleic acid amplification tests and routine partner treatment, the earlier identification and treatment of gonococcal infections could enhance gonorrhea control [41, 42].

### Increased PrEP uptake is an opportunity to increase STI screening

Although current CDC guidelines recommend routine and repeat screening for syphilis, and behaviorally based screening for gonorrhea and chlamydia, the rate of screening remains low [43, 44]. The vast majority of STIs are asymptomatic at pharyngeal and rectal sites, and studies suggest that more than 70 % would be missed with urogenital urine-based screening alone [17, 44, 45]. Many patients are not offered routine extragenital screening, even when seen at an STI clinic, and a recent study of HIV care providers showed that despite recommendations, routine STI screening was dismal [46]. Cited barriers included provider time constraints, difficulty obtaining a sexual history, language and cultural barriers, and concerns regarding patient confidentiality [46, 47].

PrEP is recommended as part of a comprehensive HIV prevention package which includes STI screening, risk reduction counseling, and condom promotion. As PrEP uptake increase, and is incorporated into various clinical settings, there is a substantial opportunity to improve STI screening frequency among high-risk MSM. Quarterly STI screening for men taking PrEP has the potential to increase the detection and treatment of STIs that would have otherwise been missed and potentially transmitted to sex partners. In an interesting observational cohort study of HIV-positive MSM, a clinic-based intervention of semiannual STI screening and risk reduction counseling reduced STI incidence in follow-up by 50 % between the 6 and 12 month visits, despite an increase in condomless anal sex with seroconcordant partners [48]. Increased STI screening coupled with enhanced partner services, including routine expedited partner therapy in MSM, may also provide an opportunity to engage sex partners for testing and treatment for further public health impact on both STI and HIV incidence.

While sexual risk compensation among PrEP users has been of concern, increased sexual risk behavior has not been observed in at least two of the PrEP clinical trials [2, 33]. PrEP uptake continues to expand among MSM and necessitates implementation of currently recommended and evaluation of new and innovative STI reduction strategies. One such strategy may be chemoprophylaxis for bacterial STIs, which could be incorporated into PrEP management. A recent pilot study by Bolan et al., demonstrated a significant reduction (Odds Ratio 0.27; 95 % CI: 0.09–0.83) in bacterial STIs among MSM randomized to receive once daily doxycycline compared to a behavioral intervention (contingency management) [49]. That STI prevention strategy is also being evaluated as a sub-study of Ipergay which has randomized study participants to event-driven prophylaxis with doxycycline (two doses of doxycycline after each sexual encounter—within at most 72 h post-event—up to a maximum of six tablets per week) vs. no doxycycline [50].

## Conclusions

PrEP holds tremendous promise to reduce further HIV incidence among MSM, with appropriate targeting and implementation, and has along with treatment as prevention become the focus of combination HIV prevention. The implementation of PrEP as part of a comprehensive HIV prevention program, which includes frequent HIV testing, early HIV treatment and viral suppression, STI screening and condom promotion, also provides a tremendous opportunity to improve STI screening, treatment, and management with subsequent benefits in sexual and reproductive health. There was a steady rise in STIs among MSM before the widespread implementation of PrEP, and the impact of the increased adoption of PrEP will be telling in the years to come. We have already witnessed a decoupling of STI and HIV incidence in some communities—increased STI incidence and lower HIV incidence—and it remains to be seen if, and how, PrEP will change these epidemiologic curves.
